# Case report: Mild encephalitis with a reversible splenial lesion associated with SARS-CoV-2 infection in a patient with *MYRF* variant

**DOI:** 10.3389/fped.2022.971432

**Published:** 2022-08-04

**Authors:** Mizuho Saito, Tomoyuki Nakazawa, Shun Toriumi, Michihiko Takasu, Hiromi Yagisawa, Yayoi Murano, Nao Miyazaki, Hirokazu Kurahashi, Akihisa Okumura, Toshiaki Shimizu

**Affiliations:** ^1^Department of Pediatrics, Toshima Hospital, Tokyo, Japan; ^2^Department of Pediatrics, Aichi Medical University, Nagakute, Japan; ^3^Department of Pediatrics, Faculty of Medicine, Juntendo University, Tokyo, Japan

**Keywords:** mild encephalitis with a reversible splenial lesion (MERS), SARS-CoV-2, COVID-19, myelin, MYRF

## Abstract

We report a 14-year-old girl with a heterozygous p. Gln403Arg variant in the *MYRF* gene, who had five episodes of encephalopathy. She experienced reduced consciousness, numbness in the arm, and impaired verbal communication from day 4 of SARS-CoV-2 infection. Magnetic resonance imaging of her head showed reduced water diffusion in the corpus callosum and deep white matter. These features were similar to those seen in her previous episodes of encephalopathy. She was treated with methylprednisolone pulse therapy and recovered completely within a week.

## Introduction

Coronavirus disease 19 (COVID-19), caused by severe acute respiratory syndrome coronavirus 2 (SARS-CoV-2), primarily causes respiratory symptoms, but various other organs are also involved. Increasing numbers of neurological complications have been reported in COVID-19 (1, 2). Neurological manifestations and complications such as stroke, cerebral venous thrombosis, seizures, meningoencephalitis, Guillain-Barré syndrome, Miller Fisher syndrome, acute myelitis, and posterior reversible encephalopathy syndrome have been reported, mainly in adults. Reports of neurological complications in children with COVID-19 are limited.

Mild encephalitis/encephalopathy with a reversible splenial lesion (MERS) is an infection-associated encephalitis/encephalopathy syndrome (3) that is common in Japanese children (4). Some cases of COVID-19-associated MERS have been reported (5–7). We previously reported a missense *MYRF* variant in recurrent MERS patients (8). *MYRF* encodes a myelin regulatory factor that is necessary for oligodendrocyte differentiation, and maintenance of mature oligodendrocytes and the myelin structure. One of the patients described in our previous report developed MERS related to a SARS-CoV-2 infection. Herein, we describe the clinical course of MERS associated with SARS-CoV-2 infection in a patient with the *MYRF* variant.

## Patient report

Written informed consent for this publication was obtained from the patient and her parent.

A 14-year-old Japanese girl developed high-grade fever and headache during the COVID-19 pandemic. A SARS-CoV-2 rapid antigen test performed at a local clinic was positive. The following day, she developed difficulty in eating, and was admitted to our hospital. Her medical history included five episodes of MERS, at the ages of 3, 6, 7, 8 and 9 years (Table 1). Three of these five episodes were associated with influenza A. Her clinical course prior to the fourth episode has been reported elsewhere, and a heterozygous p.Gln403Arg variant of *MYRF* had been identified (8). There had been a few infectious events that were not associated with encephalopathy apart from the episodes of MERS. Neurological symptoms, including unconsciousness, convulsions, and numbness in the limbs, had developed 2–4 days after the onset of fever. She had completely recovered from a previous episodes within a week.

**Table 1:** The clinical manifestations of MERS of the patient.

**Age (years)**	**Etiology of infection**	**The interval between the onset of fever and neurological symptoms (days)**	**Neurological symptoms**	**CSF analysis**			**EEG findings**	**Treatment**
				**Cell counts (/μL)**	**Protein (mg/dL)**	**MBP (pg/mL)**		
3	Unknown	3	Unconsciousness, one seizure	2	14.3	N/A	Focal slowing	Methylprednisolone pulse therapy
6	Influenza A	2	Unconsciousness	N/A	N/A	N/A	Generalized slowing	Dexamethasone
7	Influenza A	2	Unconsciousness, one seizure	5	30.1	N/A	No abnormalities	Dexamethasone
8	Influenza A	3	Unconsciousness, one seizure	N/A	N/A	N/A	N/A	Dexamethasone
9	*Mycoplasma pneumoniae*	4	Unconsciousness, numbness in the arms	N/A	N/A	N/A	N/A	Dexamethasone
14	SARS-CoV-2	4	Unconsciousness, numbness in the arms	9	25	<40	N/A	Methylprednisolone pulse therapy

*CSF, cerebrospinal fluid; MBP, myelin basic protein; N/A, not assessed*.

**Figure 1:**
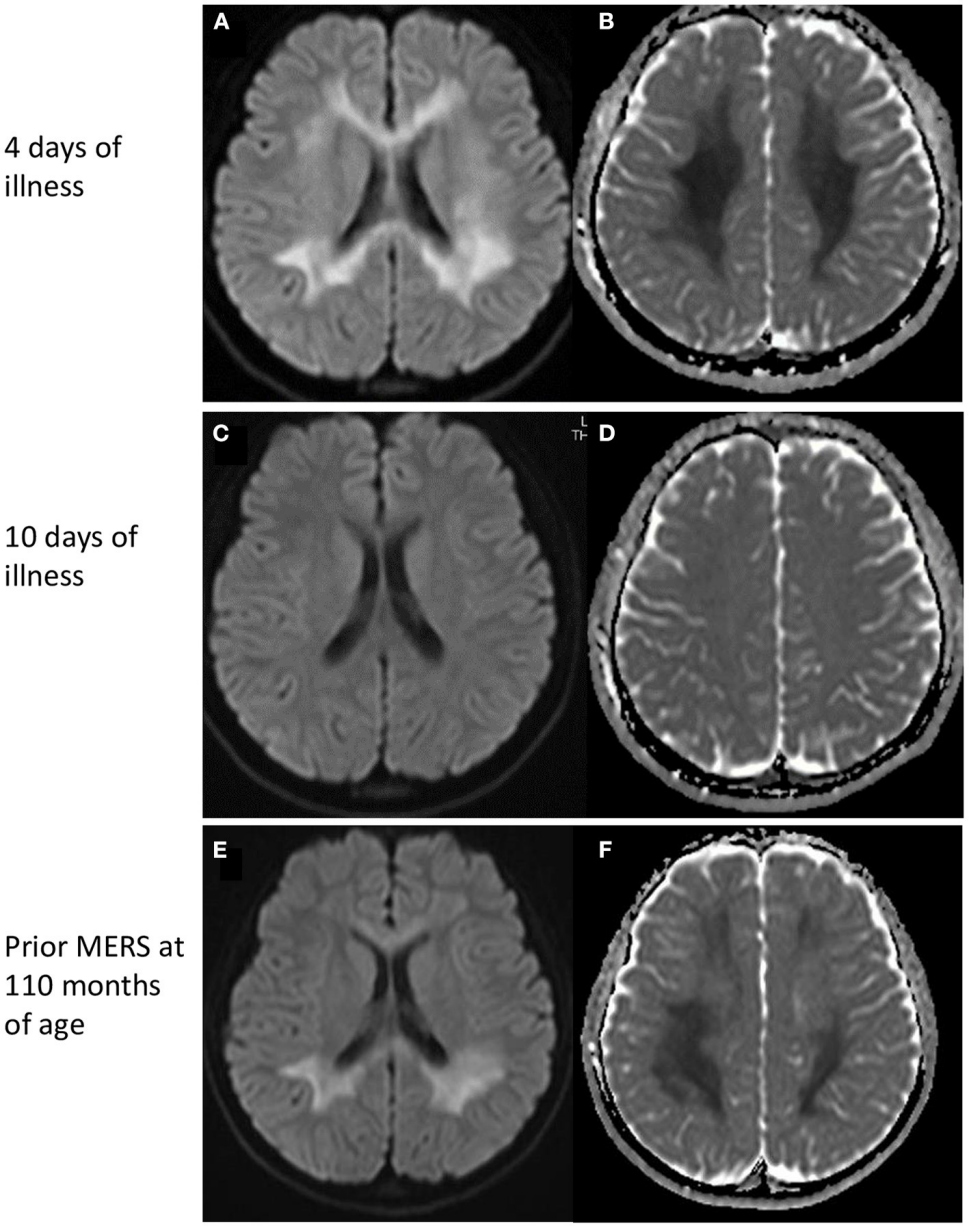
MRI findings. (Top) MRI on day 4 of illness. Restricted water diffusion was observed in the corpus callosum and deep white matter. **(A)** Diffusion-weighted images, and **(B)** apparent diffusion coefficients map. (Middle) MRI on day 10 of illness. No MRI abnormalities were seen. **(C)** Diffusion-weighted images, and **(D)** apparent diffusion coefficients map. (Bottom) MRI of a prior MERS episode at 110 months of age. A similar pattern of restricted water diffusion is evident in the corpus callosum and deep white matter. **(E)** Diffusion-weighted images, and **(F)** apparent diffusion coefficients map. MERS, Mild encephalitis/encephalopathy with a reversible splenial lesion.

On admission, she had pyrexia of 37.8°C. All other vital signs were within the normal range. Physical examination was unremarkable, apart from slight erythema of the pharynx. Her level of consciousness was normal and there was no neck stiffness. Laboratory data were unremarkable except for mild hyponatremia (134 mEq/L). SARS-CoV-2 infection was diagnosed by polymerase chain reaction (PCR) test of a nasal swab. PCR of cerebrospinal fluid was not performed. Her headache gradually improved with supportive treatment; however she complained of numbness in her arms. On the fourth day after the onset, she developed generalized spasticity, speech disturbance, and had a fixed gaze eyes wide open. Smooth pursuit was preserved. She appeared to understood the spoken word, but was unable to speak herself. These manifestations were similar to those of her previous episodes of MERS (Table 1). Brain magnetic resonance imaging (MRI) showed restriction of water diffusion in the entire corpus callosum (CC) and deep white matter (Figure 1). Cerebrospinal fluid (CSF) examination showed a cell count of 9/μL, a normal protein level (0.25 g/L), and a glucose level of 0.6 g/L. Myelin basic protein (MBP) in the CSF was below the limit of quantitation. A diagnosis of MERS was established on the basis of these findings. She was treated with a single course of intravenous methylprednisolone pulse therapy (1 g/day for 3 days), which resulted in an immediate improvement in her symptoms. A follow-up MRI on the 10th day after onset demonstrated resolution of the previously noted abnormalities. She was discharged on the 11th day after onset with no neurological sequelae. Although no formal examination of intellectual and behavioral function has not been performed, we confirmed that her school performance was average and had not been worsened until the last follow-up.

## Discussion

There have been a few reports of MERS associated with SARS-CoV-2 infection in children (5, 9, 10). MIS-C was often observed in these patients, whereas Gaur et al. reported a 9-year-old boy without MIS-C had lesions in the entire CC and centrum semiovale (9). Neurological symptoms were not very severe, including hallucinations, agitation, and disorientation. In all of the children, the MRI abnormalities disappeared within a week, and no neurological sequelae were recognized. The clinical manifestations and the time course of the MRI lesions in our patient were similar to previous reports, although reduced water diffusion was more extensive in our patient.

A widely accepted hypothesis is that an inflammatory process involving cytokines triggers glutamate accumulation in the extracellular space, resulting in cytotoxic edema, in particular of astrocytes, in MERS patients (11). However, the exact pathogenesis of MERS is not fully understood. MERS has been reported in children with Kawasaki disease (12) and urinary tract infections (13), conditions that involve inflammatory responses. In relation to COVID-19, MERS has been reported in children (5, 9, 10) and adults with MIS-C (6, 7). In addition, of the 27 children with COVID-19 pediatric MIS-C, 4 had new-onset neurological symptoms, such as encephalopathy, headaches, brainstem and cerebellar signs, muscle weakness, and reduced reflexes. Brain MRI showed splenium signal changes in all 4 children (14). MIS-C is associated with an exaggerated innate and adaptive immune response, including neutrophilia, lymphopenia, high levels of interferon-γ, and low naive CD4+ T-cell counts, with a high proportion of activated memory T cells (15). In our patient, inflammatory responses were milder than those seen in patients with MIS-C, but caused cytotoxic edema in the entire CC and cerebral white matter. This may have been related to the vulnerability of myelin maintenance associated with the *MYRF* variant. A luciferase assay revealed diminished transcriptional activity of the N-terminal region of *MYRF* by introducing the p.Gln403Arg variant, found in this patient (8).

A remarkable feature of our patient was the frequent recurrence of MERS, followed by complete recovery. Clinical manifestations were similar among the MERS episodes: the interval between fever onset and neurological symptoms was 2–4 days; CSF analysis showed subtle pleocytosis and normal protein levels; MRI demonstrated reduced water diffusion in the entire CC and deep white matter, which resolved within 1 week, and no neurological sequelae (Table 1). These facts indicate that similar pathogeneses may be related to MERS development regardless of the causative pathogen. It is noteworthy that MBP levels in the CSF were not elevated in our patient despite extensive white matter lesions. MBP is a biomarker of myelin damage that has been detected in the CSF in various conditions, including multiple sclerosis, acute disseminated encephalomyelitis, encephalitis, and acute cerebral infarctions (16). The lack of MBP elevation in CSF in our patient implies that myelin damage, if present, was subtle. This may have been related to the complete recovery despite the extensive white-matter abnormalities.

In summary, we report a patient with a missense MYRF variant, who experienced a fifth MERS episode in association with SARS-CoV-2 infection. Neurological complications of COVID-19 are not fully understood. Genetic background such as missense variant in MYRF is not a universal phenomenon. Physicians should consider genetic background in patients with MERS with SARS-CoV-2 infection, especially when they developed MERS in the past or they had no clinical features of MIS-C. Further accumulation of COVID-19 patient data associated with neurological disorders may clarify their features and treatment.

## Data availability statement

The raw data supporting the conclusions of this article will be made available by the authors, without undue reservation.

## Author contributions

MS, TN, ST, MT, HY, YM, and NM contributed to data curation. TN, HK, and AO contributed to conceptualization. AO contributed to validation for the manuscript. All authors contributed to the article and approved the submitted version.

## Funding

This work was supported by the grants from the Ministry of Health, Labor, and Welfare (21FC1005) and the JSPS KAKENHI (18K07890 and 21K07810).

## Conflict of interest

The authors declare that the research was conducted in the absence of any commercial or financial relationships that could be construed as a potential conflict of interest.

## Publisher's note

All claims expressed in this article are solely those of the authors and do not necessarily represent those of their affiliated organizations, or those of the publisher, the editors and the reviewers. Any product that may be evaluated in this article, or claim that may be made by its manufacturer, is not guaranteed or endorsed by the publisher.
