# Supplementary material for: Case report: Mild encephalitis with a reversible splenial lesion associated with SARS-CoV-2 infection in a patient with MYRF variant
Source: Front Pediatr. 2022 Aug 4;10:971432. doi: 10.3389/fped.2022.971432 (PMC9386134; doi:10.3389/fped.2022.971432)
Supplement: Supplementary file 1 [file Table_1.docx]

Supplementary Table

Details of references about MERS with SARS-CoV-2

| Reference number | Number of cases, age, sex | Neurological symptoms | Concurrence of MIS-C | Treatment | Prognosis |
| --- | --- | --- | --- | --- | --- |
| 5-1 | 10-year-old boy | Agitation, disorientation personal change | Yes | IVIG, oral prednisone | Good |
| 5-2 | 11-year-old girl | Agitation, personal change | Yes | IVIG, methylprednisolone pulse | Good |
| 6 | 75-year-old male | Dysmetria, Ataxia | No | Methylprednisolone pulse | Died due to respiratory failure |
| 7 | 60-year-old male | Vertigo, Headache, Unconsciousness | No | Analgesic drugs | Good |
| 9-1 | 12-year-old boy, | Unconsciousness | Yes | IVIG, Anakinra (Interleukin-1 antagonist), infliximab, and steroids | Good |
| 9-2 | 9-year-old boy | Altered mental state | No | None | Good |
| 10 | 13-year-old girl | Auditory hallucination, agitation, combativeness | Yes | IVIG | Good |

IVIG: Intravenous immunoglobulin
